# The Relationship between Intestinal Motility and Interstitial Cells of Cajal in Nonalcoholic Fatty Liver Mice

**DOI:** 10.5812/hepatmon.13674

**Published:** 2013-10-01

**Authors:** Ming-Liang Lu, Hua Huang, Li-Ming Liu, Jiang Chang

**Affiliations:** 1 Department of Gastroenterology, The Second Affiliated Hospital of Kunming Medical University, Kunming, China

**Keywords:** Nonalcoholic Fatty Liver, Interstitial Cells of Cajal, Intestinal Motility

## Abstract

**Background:**

Nonalcoholic fatty liver disease (NAFLD) is the most common cause of chronic liver disease in western world. However, NAFLD shows an increasing trend in China every year, which has attracted the attention of national health authorities. The previous studies have shown that NAFLD caused severe gastrointestinal motor disorders, but little is known about the interstitial cells of Cajal (ICC) role in gastrointestinal motor disorders.

**Objectives:**

The aim of this study was to observe the ICC in jejunum of nonalcoholic fatty liver mice by immunohistochemistry and assessed the relationship between intestinal motility and ICC.

**Materials and Methods:**

Thirty five Sprague-Dawley (SD) rats were randomly divided into nonalcoholic fatty liver (n = 25) and control groups (n = 10), rats were housed individually in cages and had free access to food and water, nonalcoholic fatty liver group was duplicated by high-fat diet (consisted of ordinary food, 20 g/kg cholesterol and 100 g/kg fat) feeding. Dextran blue-2000 was used to monitor the intestinal motility. The proximal small intestine was harvested to investigate the C-kit positive ICC. The hepatic tissue slices were used for pathological observation.

**Results:**

Nonalcoholic fatty liver disease was successfully established. The intestinal motility in nonalcoholic fatty liver group (49.5 ± 10.9) was weaker compared to the control group (57.3 ± 8.9), P < 0.05. The rate of ICC also have shown statistically significant differences between nonalcoholic fatty liver (4.87 ± 2.97/mm ^2^) and control groups (6.54 ± 3.13/mm ^2^), P < 0.05.

**Conclusions:**

ICC may be related to the intestinal motility in nonalcoholic fatty liver mice.

## 1. Background

Nonalcoholic fatty liver disease (NAFLD) is the most common cause of chronic liver disease in western world (1). However, NAFLD showed an increasing trend in China every year, which has attracted the attention of national health authorities. NAFLD contains simple nonalcoholic fatty liver disease, nonalcoholic steatohepatitis (NASH) and eventual fibrosis, which can be resulted in end-stage liver disease and hepatocellular carcinoma. There are no specific clinical symptoms, such as fatigued, abdominal distension, upper abdominal pain, etc. A recent study in United States (2) reported that people with NAFLD experienced an average of 12 symptoms, including abdominal pain, emotional function and systemic symptoms (bodily pain, shortness of breath (dyspnea), muscle cramps and itching). Interstitial cells of Cajal (ICC) were founded and considered as the end cells of the sympathetic nervous system by Ramon y Cajal (3), which are the pacemaker in gastrointestinal motility. A new study verified the ICC integrate excitatory and inhibitory neurotransmission with intestinal slow-wave activity to orchestrate peristaltic motor activity of the gut and impairment of the function of ICC caused severe gastrointestinal motor disorders (4). Previous studies (5, 6) have shown that NAFLD increased the oro-cecal transit time and decreased small intestinal motilities. Little is known about the exact cellular mechanism of neuronal signal transduction to smooth muscle cells in the gut. We detected ICC in jejunum of nonalcoholic fatty liver mice by immunohistochemistry and the relationship between intestinal motility and ICC was assessed.

## 2. Objectives

NAFLD follows an increasing trend in China every year. Previous studies have shown that NAFLD caused severe gastrointestinal motor disorders, but little is known about the ICC important role in gastrointestinal motor disorders. In this study we observed the ICC in jejunum of nonalcoholic fatty liver mice by immunohistochemistry and the relationship between intestinal motility and ICC was assessed.

## 3. Materials and Methods

### 3.1. Materials

Thirty five Sprague-Dawley (SD) rats, 6 - 8 weeks old and 160 – 210 g, were supplied from animal experiment center of Kunming Medical University. Rats were individually housed in cages and had free access to food and water. High-fat diet (7, 8) is consisted of ordinary food, 20g/kg cholesterol and 100g/kg fat. Dextran blue-2000 and tape were used to monitor the intestinal motility. Anti-Human CD117 (Serial number: MAB-0345) and Poly-HRP-Anti Mouse/Rabbit IgG (Serial number: KIT-9901/9902/9903) were purchased from Fuzhou Maixin Biotechnology Development Co., Ltd. The study was conducted according to the guidelines of animal research and approved by the Ethical Committee of the Second Affiliated Hospital of Kunming Medical University, with the registration number is 2013001.

### 3.2. Establishment of Animal Mode

Thirty five SD rats were randomly divided into nonalcoholic fatty liver group (n = 25) and control group (n = 10). Rats in nonalcoholic fatty liver were fed with high-fat diet and in control group were fed with ordinary diet for 12 weeks. Hepatic steatosis is defined as the presence of intracellular fat in more than 5% of hepatocytes (9).

### 3.3. Intestinal Motility Measurement

At the end of the 12th week, all modes were successfully established. All rats were fasted for 12 hours before gavage administration with dextran blue -2000 (0.4 mL). After 20 minutes, all rats received ether inhalation anesthesia and laparotomy. We measure (A) which represents the length of Dextran blue-2000 passed from pylorus sphincter to pigment the forefront and (B) that represents the total length of the small intestine, A/B × 100% indicates the intestinal motility (10).

### 3.4. Immunohistochemistry

Immunohistochemical analysis was used to investigate the c-kit expression in rats. According to protocol for immunohistochemistry, paraffin-embedded blocks were sectioned into about 4 μm thick sections. these sections were deparaffinized with xylene and rehydrated using an alcohol gradient. After microwave pretreatment in citrate buffer (pH 6.0) for antigen retrieval, sections were treated with 3% hydrogen peroxide in methanol to block endogenous peroxidase activity. Sections were incubated in 1% bovine serum albumin to block the nonspecific bindings and then incubated overnight at 4℃ with Anti-Human CD117 (Fuzhou Maixin Biotechnology Development Co., Ltd). Phosphate buffer solution (PBS) was used as a negative control. After rinsing 3 × 3 minutes with PBS, tissue sections were treated with Poly-HRP-Anti Mouse/Rabbit IgG (Fuzhou Maixin Biotechnology Development Co., Ltd) for 30 minutes at room temperature. All tissue sections were then counterstained with hematoxylin, then dehydrated, and mounted. Brown ICC membrane represents as positive result.

### 3.5. Data Extraction

The results of immunostaining were independently reviewed by two observers. Five fields (× 400 magnification) per tissue section, were randomly chosen and counted. Staining intensity was graded as 0, no staining; 1, weak staining, light yellow; 2, moderate staining, yellowish brown and 3, strong staining, brown. All 1, 2 and 3 defined as positive cells. Cajal positive cells and the observed area (mm^2^) ratio defined as the positive rate of ICC. Liver HE staining and immunohistochemistry sections were scanned by Olympus camera and the digital images were stored using the HPIAS-1000 high definition color pathology graphic analysis system, and then analyzed by image analysis system software.

### 3.6. Data Analysis

All statistical analyses were performed using SPSS 17.0 software. Continuous variables were described by mean ± standard deviation. Student's t-test was used to compare the intestinal motility of nonalcoholic fatty liver group and control group, and also compare the positive rate of ICC of nonalcoholic fatty liver group and control group in this study. All reported P values were two-sided and P < 0.05 was considered as statistically significant.

## 4. Results

**Figure 1.**
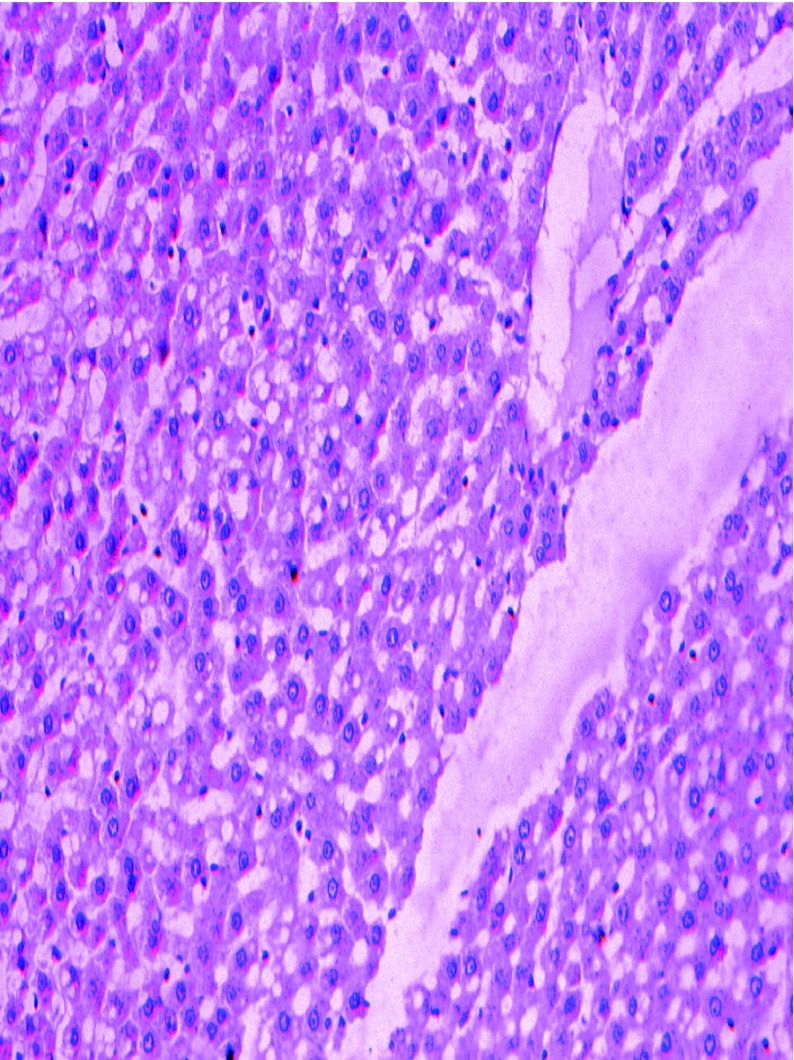
Pathological Changes of Liver After 12 weeks of High Fat Diet Hepatocyte ballooning, mixed lymphocytic and neutrophilic inflammatory infiltrate in perivenular areas, HE × 200

**Figure 2.**
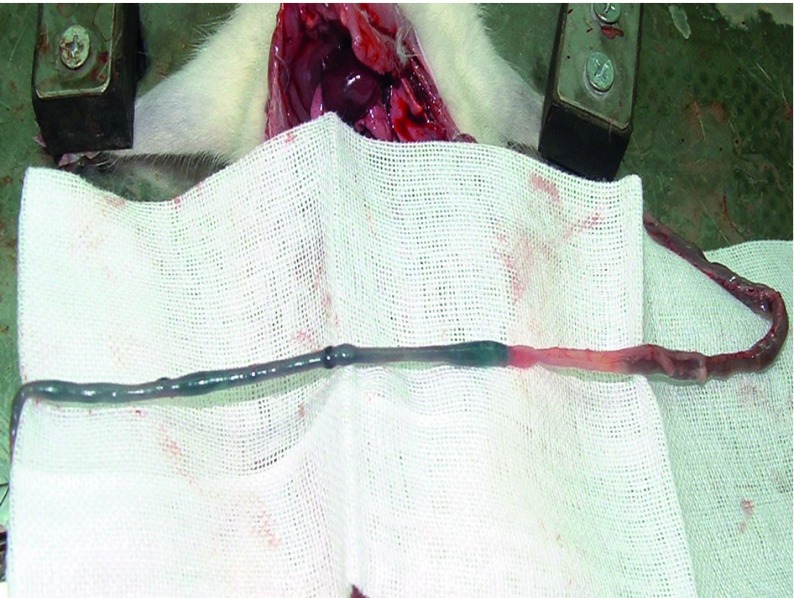
The Intestinal Motility Measurement

**Figure 3.**
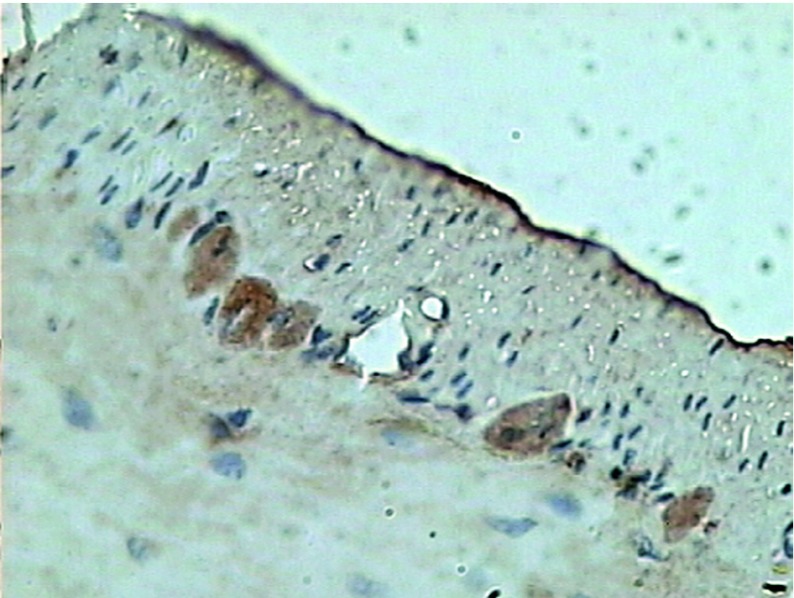
Expression of ICC in Control Group

**Figure 4.**
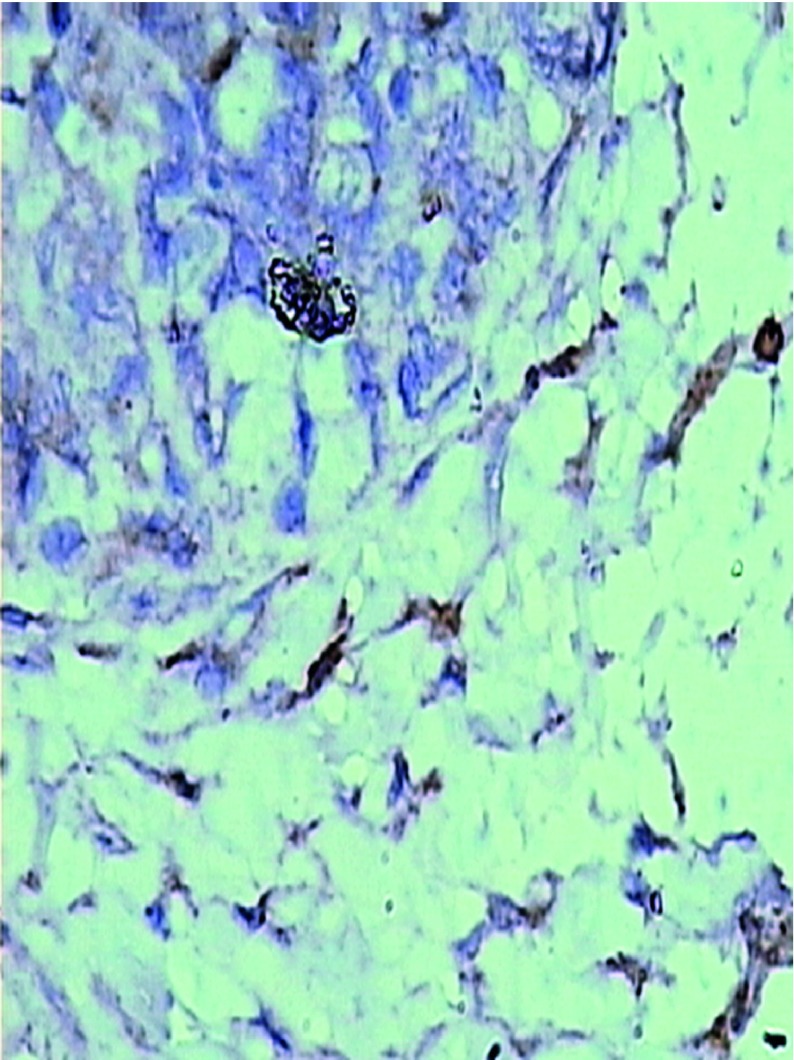
Expression of ICC in Nonalcoholic Fatty Liver Group

The pathologically nonalcoholic fatty liver mode has been successfully established (Figure 1). The intestinal motility of nonalcoholic fatty liver group (49.5 ± 10.9) weakened compared to control group (57.3 ± 8.9), P < 0.05 (Figure 2). Brown ICC membrane was recognized as a positive result. ICC mainly located in the circular and longitudinal muscle layers. C-kit positive ICC also have shown statistically significant differences between nonalcoholic fatty liver (4.87 ± 2.97/mm ^2^) and control groups (6.54 ± 3.13/mm ^2 ^), P < 0.05 (Figure 3, Figure 4).

## 5. Discussion

Nonalcoholic fatty liver disease (NAFLD) is the most common cause of chronic liver disease in the worldwide. The prevalence of NAFLD is estimated to be 30 % to 50 % (11-13). Steatosis and steatohepatitis are associated with obesity (14). Previous study has confirmed that ICC plays a crucial role in the mechanism of gastrointestinal motor disorders (15), but little is known about the ICC important role in gastrointestinal motor disorders of patients with NAFLD. This study reported the ICC in jejunum of nonalcoholic fatty liver mice and assessed the relationship between intestinal motility and ICC.

In our study, we found that intestinal motility was weaker than the control and the ICC number also decreased, which indicate that the weakened intestinal motilities may be related to the ICC. Recent studies have shown that ICC integrate the excitatory and inhibitory neurotransmission with intestinal slow-wave activity to orchestrate peristaltic motor activity of the gut (4), consisted of both myenteric (inter­muscular) plexus and submucosal plexus (16). Two studies (5, 6) demonstrated that NAFLD can be resulted in gastrointestinal motor disorders, and mainly decrease the motility. However, more evidences indicate that ICC generally was influenced by endogenous agents, such as, neurotransmitters, hormones, and paracrine substances modulate GI tract motility (17-21). Wu WC et al. (6) has found a significant increase in the number of *E. coli*, widely known source of endotoxin, in the proximal small intestinal flora of NASH group. Possible mechanism is that endotoxin might impair the ICC and thus affect the intestinal motility. At the same time, high fat diet increases the concentration of blood low density lipoprotein, which may damage the ICC due to the cytotoxicity. Overall, our data suggest that ICC may be related to the intestinal motility in nonalcoholic fatty liver mice. The further direction might perform a clinical trial to identify the relationship between ICC and intestinal motility in NAFLD.
